# The New Treatment of Hemorrhage

**Published:** 1899-04

**Authors:** 


### Abstracts and Translations.

### THE NEW TREATMENT OF HEMORRHAGE.

THERE are certain hemorrhages which, even though they are profuse, can be readily controlled because they occur in places where mechanical means can be employed for their arrest; but there are other hemorrhages which spring from vessels so deeply situated that compression and ligature do not suffice to control them, and in their presence the physician only too frequently finds himself unable to do much for his patient. In this connection the papers which have been recently published in French literature concerning the value of gelatin and calcium chloride or gelatin and sodium chloride for the purpose of causing coagulation of the blood are of interest, and some of them have been accorded space in our Progress columns. One of the most recent of these papers dealing with the indications and contraindications to hæmostasis caused by the action of gelatin is that of Paul Carnot in *La Presse Médicale* of November 16, 1898. After calling attention to the fact that he made his first communication concerning this subject in 1896, he goes on to mention the hypodermic injection of sterilized gelatin solutions for the purpose of increasing coagulability of the blood in general. He then points out that the local use of these solutions is exceedingly valuable in controlling capillary or oozing hemorrhage where compresses fail to produce the results desired, and that this substance often suffices when preparations of the iron and the acids fail. It is, of course, absolutely essential that the solution when it is injected shall be absolutely aseptic. Very commonly the gelatin has been dissolved in ordinary sea-water which has been filtered and sterilized. In other instances it is, as we have already indicated, dissolved in ordinary water to which calcium chloride has been added, calcium chloride having great power, as first pointed out by Wright, of Netley, England, in increasing the coagulability of the blood. The solution that Carnot has employed among others is one composed of gelatin, 12 drachms; chloride of calcium, $2\frac{1}{2}$ drachms; and water 1 quart. Queyat has modified this to the extent of adding a small quantity of glycerin to the solution. Any advantages this glycerin may have as a solvent are, we think, more than counterbalanced by its physiological action, which ought really to contraindicate its introduction into a mixture designed for subcutaneous injection.

As the solubility of the gelatin is a good deal increased by the application of heat, and as heat also aids in making it fluid, it is well to sterilize it immediately before it is to be used, and then to employ it before it becomes thickened by cooling, care being taken, of course, that it is not used so warm as to cause damage. It is claimed that when from one to two ounces of this solution is given under the skin into the loose subcutaneous tissues of the back or thighs it acts very speedily in causing coagulation at the bleeding point. When gelatin solutions are applied to exposed bleeding surfaces, care should be taken to protect these areas lest putrefactive changes take place in the gelatin after it is applied, and if the gelatin solutions are used in the nasal cavities to stop hemorrhage, such precautions must be carried out. Carnot then goes on to point out that there is some danger of producing hypercoagulability of the blood if the gelatin solutions are used too freely, and this possibility is to be considered as an argument against its too free employment. Indeed, Carnot believes that the free injection of both the gelatin and calcium chloride in the presence of pressing hemorrhage may, though it controls the hemorrhage, ultimately exert a deleterious influence upon the blood in general. In his opinion, therefore, the subcutaneous use of this mixture has certain disadvantages, and ought not to be commonly resorted to. He thinks that the gelatin solutions are of the greatest benefit when applied locally, and that when it is necessary to give a hæmostatic hypodermically calcium chloride itself should be employed, as under these circumstances it is effective in aiding in the coagulation of the blood, but is not capable of causing hypercoagulability, since, as is well known, calcium chloride, when given beyond a certain point, ceases to increase the coagulability of the blood and rather tends to exercise an opposite influence.

In this connection Deguy tells us, in the *Journal des Practiciens* of November 12, 1898, that the subcutaneous injection of gelatin solutions is capable of producing the following disagreeable symptoms: A condition of fever may develop, ranging from two to three degrees above normal, and this may last for a day or two. It is apt to be present in the evening and not in the morning, and sometimes is accompanied by chills and insomnia. The local accidents which follow its injection may be divided into three parts,—pain, due to the injection, of a burning character which is increased by pressure; second, a diffuse redness of the skin or pseudo-inflammatory process, violaceous in appearance, which disappears for a moment on pressure and then immediately returns; third, a diffuse induration of the tissues, having very much the same sensation as the induration due to anthrax. Usually this lasts a number of days. From what has been said for and against this method of using gelatin it is evident that it may prove to be not so valuable a hæmostatic as we were led to believe when its usefulness was first suggested; or rather, to express it otherwise, the untoward effects of this treatment may more than counterbalance the good which it is capable of doing.—*The Therapeutic Gazette.*

